# Expanding the view on the evolution of the nematode dauer signalling pathways: refinement through gene gain and pathway co-option

**DOI:** 10.1186/s12864-016-2770-7

**Published:** 2016-06-27

**Authors:** Aude Gilabert, David M. Curran, Simon C. Harvey, James D. Wasmuth

**Affiliations:** Department of Ecosystem and Public Health, Faculty of Veterinary Medicine, University of Calgary, Calgary, Canada; Biomolecular Research Group, School of Human and Life Sciences, Canterbury Christ Church University, Canterbury, UK; Current address: MIVEGEC (UMR CNRS/IRD/UM 5290), Montpellier, France

## Abstract

**Background:**

Signalling pathways underlie development, behaviour and pathology. To understand patterns in the evolution of signalling pathways, we undertook a comprehensive investigation of the pathways that control the switch between growth and developmentally quiescent dauer in 24 species of nematodes spanning the phylum.

**Results:**

Our analysis of 47 genes across these species indicates that the pathways and their interactions are not conserved throughout the Nematoda. For example, the TGF-β pathway was co-opted into dauer control relatively late in a lineage that led to the model species *Caenorhabditis elegans*. We show molecular adaptations described in *C. elegans* that are restricted to its genus or even just to the species. Similarly, our analyses both identify species where particular genes have been lost and situations where apparently incorrect orthologues have been identified.

**Conclusions:**

Our analysis also highlights the difficulties of working with genome sequences from non-model species as reliance on the published gene models would have significantly restricted our understanding of how signalling pathways evolve. Our approach therefore offers a robust standard operating procedure for genomic comparisons.

**Electronic supplementary material:**

The online version of this article (doi:10.1186/s12864-016-2770-7) contains supplementary material, which is available to authorized users.

## Background

An animal’s phenotype, whether developmental or behavioral in response to stimuli, is mediated through signalling pathways. While there are a limited number of pathway types, they can vary in terms of membership and can be strung together to form complex interactions [1]. Given their role in development and in human disease, understanding how these pathways evolve is an important, outstanding question. The surge of metazoan genomes has created the temptation to search for orthologous genes involved in a pathway of interest and then to offer some biological interpretations. Such comparisons are a routine feature of publications announcing new genomes. However, genomes are often published at a relatively early draft stage, with errors in the assembly leading to incorrect or absent gene models. This is an important pitfall for comparative genomics and molecular biology analyses; the non-detection of an orthologue is not necessarily biologically relevant, but can be the consequence of technical issues. Here, we have investigated the utility and viability of genomic comparisons in the context of the evolution of signalling pathways. As a model pathway, we have analyzed the conservation of the *Caenorhabditis elegans* dauer larva development pathways across the Nematoda.

**Fig. 1:**
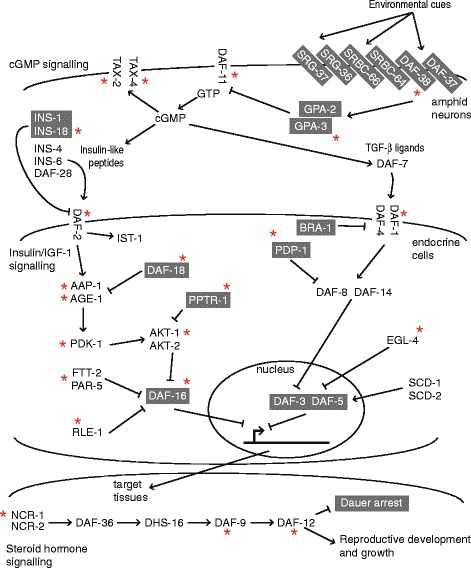
Summary of the signalling pathways known to control the switch between dauer and growth in *C. elegans*. Genes in a grey box are those whose function promotes the formation of the dauer stage. The function of the other genes leads to reproductive development. The red asterisk marks genes that are conserved in at least Clades III, IV and V

The free-living nematode *C. elegans* is one of the most studied animals and has a genome assembly of the highest quality [2]. As a consequence, its signalling pathways are generally well-known and well-characterized. During the larval development of *C. elegans*, a decision occurs in the first larval stage (L1), which involves three environmental factors: population density, food supply and temperature. In replete conditions, development continues through the L2, L3 and L4 molts to adult. In response to environmental stress, the animal enters an arrested developmental stage [3]. Termed dauer, the stage is long-lived and is exited once conditions improve. The regulation of signal transduction from stimuli to developmental decision involves four pathways: cGMP, Insulin/IGF-1, TGF-β and steroid hormone (dafachronic acid) synthesis [4] (Fig. 1).

The phylum Nematoda is an excellent system for the study of pathway evolution for two reasons. Firstly, from a free-living ancestral state, parasitism of plants and animals has evolved independently at least 15 times [5]. These transitions were made possible through a range of molecular adaptations [6], which must include changes in the architecture of signalling pathways as they respond to new stimuli [1]. Secondly, constituents of these pathways are likely a promising source of new anti-parasitics, possibly through repurposing already licensed drugs [7]. Multi-drug resistance is a growing problem in veterinary medicine and an emerging threat to the efficacy of human-based mass drug administration programs [8]. The search for new control strategies has motivated sequencing projects for many species of parasitic nematodes [5]. Morphological and behavioral similarities between the *C. elegans* dauer and the infective stage of some parasitic species has led to the dauer hypothesis, in which pre-adaptations in free-living ancestors led to the multiple independent transitions to parasitism as a life strategy [9, 10]. This hypothesis is gaining support through biochemical manipulation of the pathways in parasitic species [11]. Understanding the conservation of genes that control this important stage could therefore prioritise targets for new anthelmintics.

Here, we have identified homologues – putative orthologues – of 47 genes across *C. elegans* and 23 other nematode species, both free-living and parasitic. While the pathways controlling the dauer transition are generally well-conserved, we identify an evolutionary path of gene duplication and pathway co-option that leads from the ancestral nematode to *C. elegans*. Further, we highlight the issues encountered and solutions implemented when working with draft genomes.

## Results

### Identifying potential homologues for dauer signalling genes

**Fig. 2:**
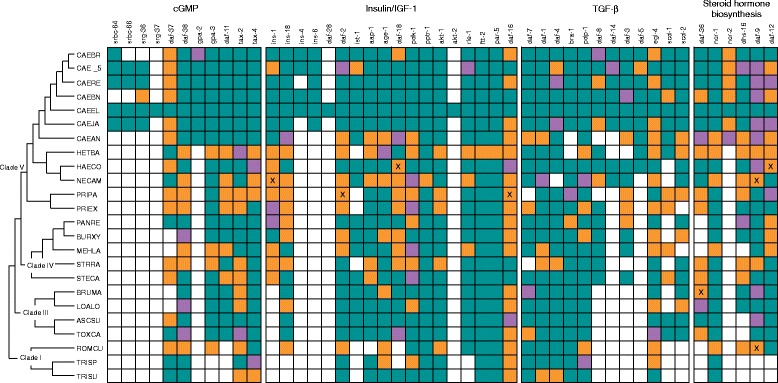
Presence and absence of homologues to *C. elegans* genes that control the dauer switch. Each box gives the best case for the homologue. A published gene model that was matched to a *C. elegans* dauer gene using fuzzy reciprocal BLAST is shown in green. Homologues detected using one of the six other search strategies and that led to minor refinements in a published gene model are shown in purple. Homologues that required major improvements to the published gene model or for which no gene model was present in the published annotation, are shown in orange. An X denotes a homologue that was detected by mapping DNA sequence reads to the *C. elegans* protein sequence. The species codes are: ASCSU – *Ascaris suum*; BRUMA – *Brugia malayi*; BURXY - *Bursaphelenchus xylophilus*; CAE_5 – *Caenorhabditis* sp. 5; CAEAN – *Caenorhabditis angaria*; CAEBN – *C. brenneri*; CAEBR – *C. briggsae*; CAEEL – *C. elegans*; CAEJA – *C. japonica*; CAERE – *C. remanei*; HAECO – *Haemonchus contortus*; HETBA – *Heterorhabditis bacteriophora*; LOALO – *Loa loa*; MELHA – *Meloidogyne hapla*; NECAM – *Necator americanus*; PANRE – *Panagrellus redivivus*; PRIEX – *Pristionchus exspectatus*; PRIPA – *Pristionchus pacificus*; ROMCU – *Romanomermis culicivorax*; STECA – *Steinernema carpocapsae*; STRRA – *Strongyloides ratti*; TOXCA – *Toxocara canis*; TRISP – *Trichinella spiralis*; TRISU – *Trichuris suis*

We used seven strategies to search the protein sequences of *C. elegans* dauer genes against the gene models and assembled genome sequences of 23 other nematode species [12–30]. For each *C. elegans* protein that seeded a search, we carried out a manual inspection for both positive and negative results. We first relied on the fuzzy reciprocal BLAST approach (FRB) [31]. Following manual inspection, FRB identified 80 % (173 of 216) homologues in *Caenorhabditis* species, and 61 % (300 of 538) for the remaining 17 nematode species (Fig. 2). In this second group, gene model accuracy was not significantly correlated (r = 0.25) with completeness of genome annotation (Additional file 1: Table S1). Across all species, the assignment of homology for 33 % of genes relied on comparisons against the raw genome assembly [32–37]. Experience of various genome analyses has shown that RNA-seq assemblies inflate gene number. A limited, formal analysis supported this position and did not lead to any additional dauer genes being identified (Additional file 2: Table S2). We observed the gamut of problematic gene models that were incorrect or missing in the genomes’ released annotations (Additional file 3: Figure S1): missing coding regions, the true gene split between multiple models, or a single model containing multiple true genes. Many cases could be corrected manually. Given the manual inspection step, we are confident to have found homologues between *C. elegans* genes and other nematode species. In a few cases, where two or more copies are found in another species, these are likely recent duplications, signifying a co-orthologous relationship with *C. elegans*. Given these duplications, the lack of gene-order conservation between *C. elegans* and other nematodes, and that performing a detailed phylogenetic reconstruction on every gene would be prohibitively time-consuming, we refer to the matches as homologues, except in specific examples. Gene models for the homologues are available in the Additional file 4: File S1 and Additional file 5: FileS2.

Homologues for 14 *C. elegans* genes, *daf-38, tax-2, tax-4*, *daf-2*, *age-1*, *pdk-1*, *pptr-1*, *akt-1*, *ftt-2*, *daf-16*, *daf-1*, *pdp-1*, *egl-4, ncr-1* were present in all 23 species (Fig. 2). If we removed species of Clade I from the count, then homologues for a further eight genes are present in all remaining species. Below, we consider each of the four pathways and the conservation of homologues.

### cGMP signalling

The genes *tax-2* and *tax-4* encode subunits of a sensory transduction nucleotide-gated channel and are conserved throughout the species examined. The *daf-11* encoded transmembrane guanylate cyclase, whose activity powers the channel, is found in Clade III, IV and V species. The set of G-protein subunits and the G-protein coupled receptors (GPCRs) are less well conserved. Their proteins are implicated as sensors of environmental cues and control *C. elegans*’ dauer. The GPCR *daf-38* is ubiquitous, with *gpa-3* lost in the *Trichinella*/*Trichuris* lineage. The other components are likely restricted to the *Caenorhabditis* species, with *srg-37* found only in *C. elegans*.

### Insulin ligands

The *C. elegans* genome is predicted to contain 40 genes that encode for insulin-like proteins [29]. Five have been implicated in the regulation of dauer [38–40]. Proteins encoded by *ins-4*, *ins-6* and *daf-28* are agonists and are restricted to *Caenorhabditis* species. The antagonists, *ins-1* and *ins-18*, are more widely conserved.

### Insulin/IGF-1 signalling

**Fig. 3:**
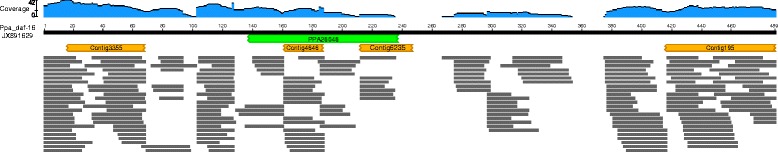
Absence of *P. pacificus daf-16* from assembled genome. The protein sequence encoded by *P. pacificus daf-16* (GenBank accession: JX891629) is represented by the black bar. The location of the best BLASTP alignment with the published *P. pacificus* gene models is shown in green. The locations of the best TBLASTN alignments with the published *P. pacificus* genome assembly are shown in orange. The horizontal grey bars are the locations the alignments with *P. pacificus* short DNA sequence reads (SRA: ERR777789), the blue histogram reports the depth of coverage at each position in *daf-16*

This pathway is broadly conserved across all species. Variability of conservation was focused around two heteromeric complexes. In the first complex, AKT-1/AKT-2, homologues to *C. elegans akt-1* were found in all species. However, *akt-2* was restricted to *C. elegans*. In the second complex, FTT-2/PAR-5, homologues for at least one of the genes were found in each species. Within both complexes, components shared high sequence similarity, indicating gene duplication events at various points in the phylum. The negative dauer regulator, *ist-1*, likely arose after the split with Clade I, with subsequent independent loss in other lineages, including that leading to *Pristionchus* species. The *P. pacificus daf-16* had been previously cloned and functionally characterized (GenBank Accession: JX891629) [41]. However, our searches of the *P. pacificus* genome assembly and associated models revealed little support for the presence of *daf-16* [17]. Aligning the short sequence reads to the *C. elegans* dauer proteins revealed a read depth and coverage consistent with other predicted *P. pacificus* homologues [37]. Alignment of the confirmed *P. pacificus daf-16* with sequence reads showed that the region of the genome containing the gene is probably misassembled (Fig. 3).

### Dauer TGF − β signalling

Broadly conserved across the phylum were genes that encode the TGF − β ligand, DAF-7, the subunits of its receptor, DAF-1 and DAF-4, and the receptor’s regulator BRA-1. Genes encoding the Smad transcription factors were increasingly restricted in their phylogenetic distribution. *daf-8,* an inducer of transcription, and *daf-3,* a repressor, were found in Clade IV and V species. Another inducer, *daf-14*, was found throughout Clade V. Homologues to another repressor, *egl-4,* were found throughout, while *daf-5* was restricted to Clade V species. In the Clade I species, we were unable to find homologues for any of the Smad transcription factors, *daf-5*, *scd-1* or *scd-2*.

### Phylogenetic reconstruction of dauer TGF-β ligand

Notable from the TGF-β pathway was the absence of a homologue for its ligand, *daf-7*, in *Strongyloides ratti*. Several studies have proposed a putative orthologue for *daf-7*; the candidate gene has been cloned from *S. ratti* (AY672707), the sister species *Strongyloides stercoralis* (AAV84743) and *Parastrongyloides trichosuri* (ABQ10586) [42, 43]. While we could not find a significant alignment between AY672707 and any *S. ratti* gene model, we did successfully recover a splice-aware alignment from the raw genome assembly [33, 34]. However, even the inclusion of this model in the *S. ratti* annotation did not generate a FRB match to *C. elegans daf-7*.

**Fig. 4:**
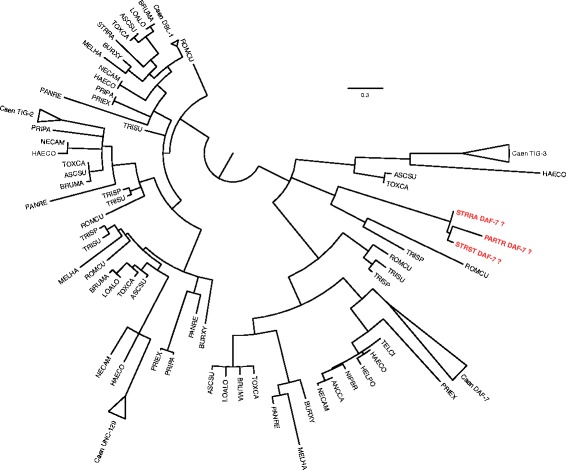
Phylogenetic reconstruction of nematode homologues to *C. elegans* TGF-β genes. Branches with less than 50 % bootstrap support have been left unresolved. In red are the three Parastrongyloides/Strongyloides sequences previously annotated as DAF-7. The species codes are as in Fig. 2, including: ANNCA – *Ancylostoma caninum*; HELPO – *Heligmosomoides polygrus*; NIPBR – *Nippostrongylus brasilensis*; PARTR – *Parastrongyloides trichosuri*; STRST – *Strongyloides stercoralis*; TELCI – *Teladordsagia circumcinta*. When monophyletic, the *Caenorhabditis* species have been collapsed and labelled Caen. The full newick format tree is in Additional file 6: File S3

There are five genes in *C. elegans* that encode annotated TGF-β ligands – *daf-7*, *dbl-1*, *unc-129*, *tig-2* and *tig-3*. Using the FRB results, we assembled the putative homologues across the 24 nematode species, to which we added individually cloned nematode TGF-β ligands [42–46]. From a protein sequence based maximum likelihood phylogenetic reconstruction, we noted that monophyletic clades were returned for DBL-1, UNC-129 and TIG-2 proteins (Fig. 4). The topologies within each clade were broadly consistent with the known species relationships [47]. If we removed the assumption that AY672707, AAV84743, ABQ10586 are DAF-7 orthologues, then we observed a monophyletic clade for DAF-7, which importantly included Clade I sequences. We also confirm the orthologous relationship between DAF-7 and ligands in *H. contortus*, *N. americanus* and *B. malayi*. The three sequences from *S. ratti*, *S. stercoralis* and *P. trichosuri* were placed with TIG-3 from *T. spiralis* and *R. culicivorax*, a clade that formed a trifurcation with TIG-3 from other species and DAF-7. The available gene expression data shows that the *Strongyloides*/*Parastrongyloides* genes more closely resemble *C. elegans tig-3*: low in L1, L2 and L3; significantly increased in the infective L3; low or undetectable in adults [42, 48]. *C. elegans tig-3* has been implicated in ageing [49, 50] and may have a role in iL3 longevity in some parasites.

### Steroid hormone signalling

This pathway was conserved throughout species in Clades III, IV and V, with *ncr-2* restricted to *Caenorhabditis* species. High sequence similarity between the sterol transport proteins NCR-1 and NCR-2 (BLASTP; Expect = 0) indicates a gene duplication event early in the *Caenorhabditis* lineage. Both the nuclear hormone receptor, *daf-12*, and the cytochrome P450 *daf-9* are essential in the regulation of dauer development [51, 52]. Searches of the gene models and genome assembly failed to return a match for *daf-12* in either *H. contortus* isolates. However, a likely candidate *daf-12* was found when *C. elegans* proteins were aligned to DNA sequence reads generated as part of the *H. contortus* genome projects [14, 15, 37]. The same approach was necessary to identify *daf-9* in *N. americanus.* Homologues to neither could be robustly identified in *T. spiralis* and *T. suis*. However, it is important to note that both these species have significantly reduced complements of *nhr* and *cyp* gene classes (D. Curran pers. comm.).

## Discussion

The ability of *C. elegans* to undergo dauer arrest is one of the most renowned postembryonic developmental transitions in any species. Here, we present the evolution of the signalling pathways that control dauer throughout the nematode phylum. There are four points for discussion: pathway-level evolution in dauer, gene-level evolution in dauer, the analogy of free-living dauer and parasitic infective stages, and the quality of draft genomes.

The decision to enter dauer is controlled in *C. elegans* by at least four pathways, with Insulin/IGF-1 and TGF-β acting synergistically. With compelling comparisons drawn between the dauer arrested state of *C. elegans* and the infective stage of some parasitic nematodes [9], the question here was whether common genetic pathways existed across the phylum that would likely control this transition. The answer is yes for species of Clades III, IV and V, and unlikely for Clade I.

Of the four pathways the cGMP and Insulin/IGF-1 pathways are broadly conserved. In the cGMP pathway, the nucleotide-gated channel (subunits: *tax-2* and *tax-4*) is ubiquitous. However, the identity of the genes involved in the detection of environmental cues, the GPCRs and G-proteins is less clear. Our approach has been to take a simple assumption and look for the homologues – putative orthologues – of *C. elegans* proteins. However, genes that sample the environment are frequently members of large gene families that display complex patterns of gene birth and death [53–55]. This makes transference of function difficult, even between closely related species, as observed in *Caenorhabditis* pheromone receptors [56]. In non-*Caenorhabditis* species, it is likely that several GPCRs are involved in sensing environmental cues. However, preliminary evidence points to a reduced complement in the parasites, *H. contortus*, *S. ratti* and *A. suum* (data not shown). Whether this is a consequence of the more restricted environments of parasitic nematodes remains to be determined. While the antagonist of the insulin receptor DAF-2 appear to be broadly conserved in nematodes, putative orthologues to known agonists in *C. elegans* were more restricted. It is probable that DAF-2 receptors in non-*Caenorhabditis* species bind other insulin-like peptides. A comprehensive genomic survey and phylogenetic reconstruction of these relatively short – approximately 110 amino acids – is necessary.

Single gene studies have proposed homology in the Insulin/IGF-1 pathway in several parasitic species, with functional assays for *daf-2* [57], *age-1* [58] and *daf-16* [41, 59–61]. The conservation of the DAF-7–TGF-β pathway is less clear. The pattern of gene homology in Clade IV and V species is indicative of a DAF-7-TGF-β pathway that acts synergistic to Insulin/IGF-1. The antagonist pair, *daf-8* and *daf-5*, arose in the MRCA of Clade IV and V. The activation and repression of this pathway with respect to the dauer phenotype has been fine-tuned with *daf-14* and *daf-5* in the MRCA of the Rhabditida (*H. contortus* and *N. americanus*) and Rhabditoidea (*Caenorhabditis*). From a broader perspective, the patterns of gene homology indicate a more fundamental function for the DAF-7 induced pathway and later co-option in the Clade IV/V MRCA to the regulation of life stage transition. Support for this hypothesis is provided by the phenotypes exhibited by mutant strains and RNA interference assays. Knock-out of the genes early in the pathway display defects related to related to egg-laying in addition to the altering likelihood of dauer development. The phenotypes associated with *daf-3* and *daf-5* are restricted to dauer. However, even within the Clade V nematodes, the expression of *daf-7* is confounding. In *C. elegans*, *daf-7* expression peaks in L2, reducing significantly in a dauer arrested animal [42]. A reduction of *daf-7* expression likely leads to dauer entry. However, inspection of the RNA-Seq data from *H. contortus* and *N. americanus* shows that *daf-7* expression is maximal in the infective L3 stage, which is supported by real-time PCR in *H. contortus* [14, 16, 46]. One proposition is that the DAF-7–TGF-β pathway has flipped in parasitic nematodes to maintain the infective stage and promotes transition to the adult [62]. An assumption there is that the role of the pathway in *C. elegans* is the ancient characteristic, with the parasitic one being derived. Interestingly, the *daf-7* orthologues of Clade III *B. malayi* and Clade I *T. suis* have expression patterns that match the Clade V parasites [26, 45], but in Clade III *T. canis* expression in the adult is significantly greater than the infective L3s (iL3) [24]. Therefore, we propose an alternative evolutionary path, in which the iL3 to adult transition through DAF-7–TGF-β mediation is basal, and the behavior observed in *C. elegans* is derived. Detailed gene expression profiles across the entire life-cycle for the free-living *P. pacificus* and *P. redivius* will, we hope, provide enlightenment.

The final pathway, steroid hormone biosynthesis, is well conserved in Clade III, IV and V species. In the final step, dafachronic acids, a class of steroid hormones generated by *daf-9*, bind to *daf-12,* a nuclear hormone receptor. For *Strongyloides stercoralis*, a facultative parasite (Clade IV), the application of exogeneous dafachronic acid to L1 worms led to the development of free-living adults, rather than iL3s [11]. The cytochrome P450 *daf-9* was previously reported to be lost in parasitic lineages [63, 64]. Searches for functional equivalents in cytochrome P450s will be difficult in such a large gene family [65]. Here, we have provided promising candidates for validation by functional rescue.

For species of Clade I, the most striking absences are for *daf-11* in all three species and *daf-9* in *T. spiralis* and *T. muris*. The *daf-11* gene encodes a receptor that defines the cGMP pathway, which controls *daf-2* transcription in response to environmental stimuli. The gene *daf-9* encodes for a cytochrome P450 that generates the sterol ligands for DAF-12, which leads to reproductive growth. The phylogenetic distribution of *daf-9* is most parsimoniously explained by gene loss, a consequence of a major reduction in genome size and gene count. Our current work shows that *T. spiralis* and *T. muris* likely only have two or three cytochrome P450 genes (unpublished). These must have broad specificity and are unlikely to carry out the precise role required of *daf-9* in *C. elegans*. Similarly, there are few other guanylate cyclases encoded in the three Clade I species to carry out the function of *daf-11*. The role of the cGMP pathway in transmitting signal to the insulin pathway through *daf-11* may therefore have arisen after the split of Clade I nematodes. Alternatively, *daf-11* may be present in other, as yet unsampled, Clade I species and has been lost in two distinct lineages. A functional analogue of *daf-11* might be unnecessary in the species surveyed. Their infective stage is L1 for *T. spiralis* and *T. suis* and L2 in *R. culicivorax*. In all these species the transition from infective larva to adult is through a series of rapid molts, the control of which may not be in response to external stimuli.

The refinement of signalling pathways transducing a stimulus to a response are often considered at a macro-level, with the co-option of new pathway types [1]; here, DAF-7–TGF-β in Clade V nematodes. Equally important are micro-level refinements. We observed gene duplications restricted to *Caenorhabditis* and to just *C. elegans*. The products interact and provide functional redundancy. There were instances of putative duplications for other genes in other nematodes, though genome assembly error through allelic polymorphism cannot be ruled out for some.

Finally, it is important to make a comment on the use of draft genome assemblies in non-model organism genomes. The assignment of gene homology between *C. elegans* and another species relies on careful consideration of sequence similarity shared between two gene models or a model and a region of an assembled genome [33–36]. In some instances, this required aligning short genomic reads against seed proteins [37]. We recognize the limitations in transferring functional annotation based on sequence similarity, but emphasize the use of multiple alignment algorithms and the manual inspection of each alignment. This gene-by-gene consideration proved critical, as no single alignment score threshold would have sufficed. Importantly, the sole reliance on gene models would have resulted in the homologous relationships for over 30 % of the genes going unfound. Central to this is the new crop of protein-to-genome aligners that are refining long established genome annotations [33, 66].

## Methods

### Genomic datasets

Genome assemblies and predicted gene models were downloaded from Wormbase [29] for all species, except *Romanomermis culicivorax*, which was downloaded from http://nematodes.org/genomes/romanomermis_culicivorax/index.html. The gene expression data for each species was taken from the supplementary information of each genome publication and is cited in the text.

### Sequence searches

Seven search strategies were used to identify potential homologues. The first is our own implementation of the fuzzy reciprocal BLASTP hits first used across 12 species of *Drosophila* [31]. The remaining six methods used *C. elegans* protein sequences to search the sequence of the genome assemblies: TBLASTN [32], Figmop [33], spaln [34] exonerate [36], genBLAST [35] and DIAMOND [37]. We have previously used Figmop to annotate cytochrome P450 genes missed by standard gene finding protocols [33]. All search results were inspected manually with the help of Kablammo [67]. Gene models generated by the six genome-based search strategies often overlapped gene models published as part of that species’ genome paper. In these instances, both gene models were aligned to the *C. elegans* protein. Our newer gene models were considered a minor improvement if their alignment had a coverage 5 % greater than the published gene model, and a major improvement if the alignment has a coverage 15 % greater. Where our predictions overlapped multiple published gene models or no gene model, the new model was considered a major improvement. The presence and absence matrix (Fig. 2) was generated using the ETE Toolkit [68]. The alignments generated by DIAMOND were processed using samtools [69] and Fig. 3 was generated using Geneious version 9 (http://www.geneious.com, [70]).

### TGF-β phylogenetic reconstruction

In addition to the putative homologues to DAF-7, searches were run using *C. elegans* DBL-1 (Wormbase: T25F10.2), TIG-2 (F39G3.8), TIG-3 (Y46E12BL.1) and UNC-129 (C53D6.2) as the seeds. Individually cloned sequences were identified from the literature. The alignment was built with MAFFT, using the linsi options [71]. The most appropriate phylogenetic model was LG + G [72]. The phylogenetic tree was reconstructed with PhyML using the following command line options: ‘-c 4 -m LG -v 0.0 -a e -o tlr -f d -d aa -b 1000 -s BEST --rand_start –n_rand_starts 101’ [73]. Nodes with less than 50 % bootstrap support were left unresolved. The final tree figure was generated using TreeGraph 2 [74] and FigTree (http://tree.bio.ed.ac.uk/software/figtree/).

## Additional files

Additional file 1: Genome assembly information for the species studied, includes: BioProjectID, assembly N50, CEGMA results, PubMedID of publication. (XLSX 14 kb) Additional file 2: Gene match results for transcriptomes from *Haemonchus contortus, Strongyloides ratti* and *Trichuris suis*. (XLSX 12 kb) Additional file 3: Examples of errors in gene models. (PPTX 888 kb) Additional file 4: Gene models in gff format. (TGZ 240 kb) Additional file 5: Gene models in fasta format. (TGZ 545 kb) Additional file 6: Newick tree format for Figure 4. (TXT 3 kb)
