# Supplementary figures and images for: Expanding the view on the evolution of the nematode dauer signalling pathways: refinement through gene gain and pathway co-option
Source: BMC Genomics. 2016 Jun 27;17:476. doi: 10.1186/s12864-016-2770-7 (PMC4924289; doi:10.1186/s12864-016-2770-7)

## Slide 1
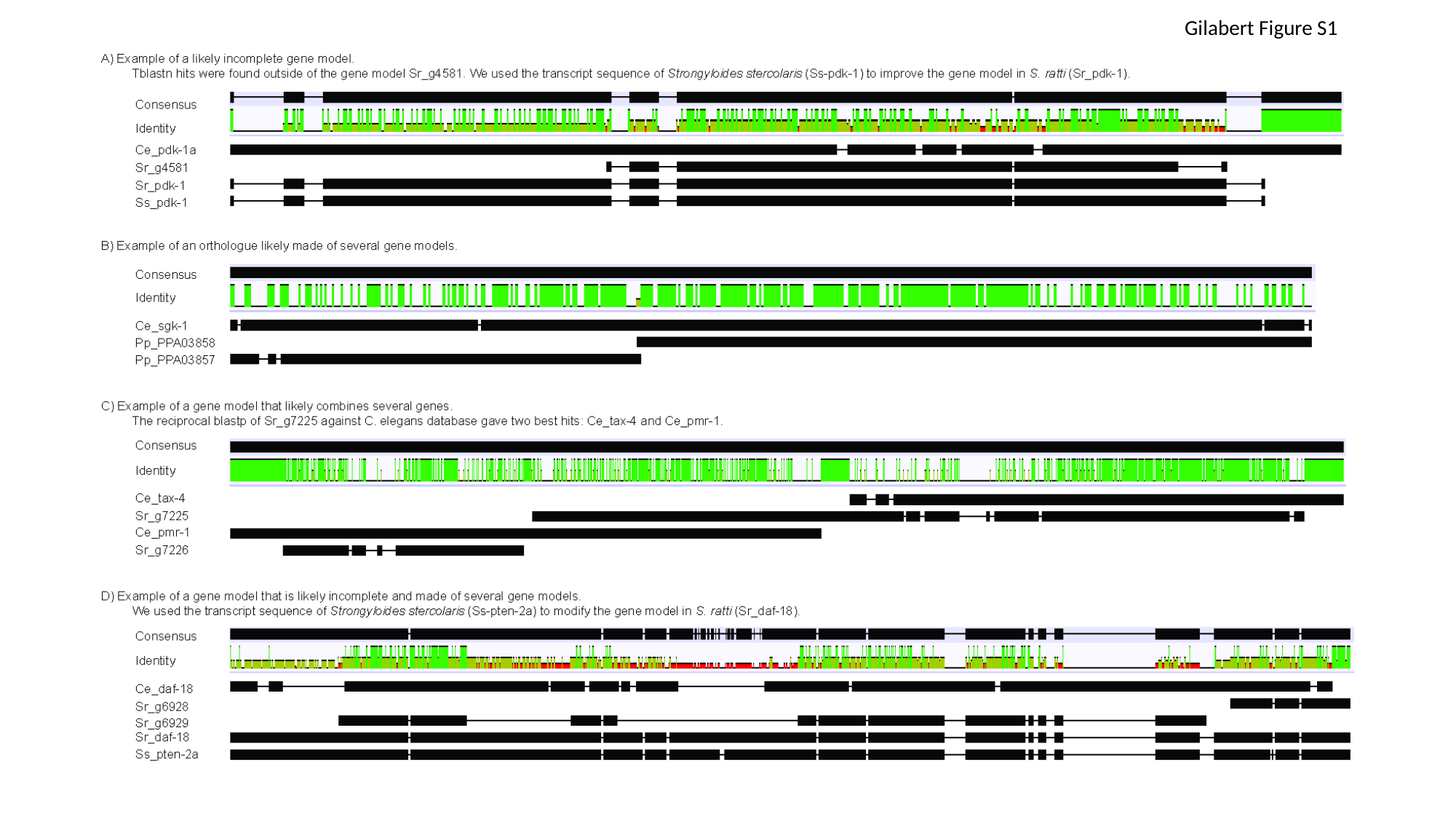

Gilabert Figure S1

Supplement: Additional file 3: — Examples of errors in gene models. (PPTX 888 kb) [file 12864_2016_2770_MOESM3_ESM.pptx]
